# Myrrh induces the apoptosis and inhibits the proliferation and migration of gastric cancer cells through down-regulating cyclooxygenase-2 expression

**DOI:** 10.1042/BSR20192372

**Published:** 2020-05-20

**Authors:** Mengxue Sun, Jie Hua, Gaoshuang Liu, Peiyun Huang, Ningsheng Liu, Xiaopu He

**Affiliations:** 1 Department of Geriatrics, Jiangsu Province Hospital and Nanjing Medical University First Affiliated Hospital, China; 2 Department of Gastroenterology, Jiangsu Province Hospital and Nanjing Medical University First Affiliated Hospital, China; 3 Department of Pathology, Nanjing Medical University, Nanjing 210000, China; 4 The Key laboratory of Antibody Technique of Ministry of Health, Nanjing Medical University, Nanjing 210000, China

**Keywords:** apoptosis, gastric cancer, Myrrh, xenograft

## Abstract

**Objective:** The present study is designed to evaluate the anti-tumor effects of myrrh on human gastric cancer both *in vitro* and *in vivo*. **Methods:** The gastric cancer cell proliferation was determined by MTT assay. Apoptosis was measured by flow cytometry and Hoechst 33342 staining. Wound healing was performed to evaluate the effects of myrrh on the migration. COX-2, PCNA, Bcl-2, and Bax expressions were detected by Western blot analysis. A xenograft nude mice model of human gastric cancer was established to evaluate the anti-cancer effect of myrrh *in vivo*. **Results:** Myrrh significantly inhibited cellular proliferation, migration, and induced apoptosis *in vitro* as well as inhibited tumor growth *in vivo*. In addition, myrrh inhibited the expression of PCNA, COX-2, and Bcl-2 as well as increased Bax expression in gastric cancer cells. **Conclusion:** Myrrh may inhibit the proliferation and migration of gastric cancer cells, as well as induced their apoptosis by down-regulating the expression of COX-2.

## Introduction

Globally, gastric cancer (GC) is the most diagnosed malignancy and the second leading cause of cancer-related death [1,2]. Despite multiple treatment options, such as surgery, radiotherapy, conventional chemotherapy, molecular, biological therapy, and targeted therapy [3,4], the prognosis of GC remains poor. Novel treatments for gastric cancer are still needed.

Myrrh, a resinous exudate of *Commiphora* family [5], has long since being used for the treatment of inflammatory diseases. Numerous pharmacological studies have investigated its anti-inflammation mechanisms. Guggulsterone, a core functional extract from myrrh, inhibits GC tumor growth in animal models and enhances the chemo-sensitivity of breast cancer cells [6,7]. In addition, *Commiphoramyrrha* induces the apoptosis of human prostatic cancer and hepatocellular carcinoma cells [8–10]. However, the efficacy of myrrh in treating GC has not been studied. The present study is the first attempt to explore the effect of myrrh on the morphology of GC cells

Cyclooxygenase (COX) is a potential target in tumor treatment and prevention [11]. In GC, COX-2 participates in tumor-associated processes, such as angiogenesis, invasion, and immune evasion, and indicates histological subtype, tumor size, and developmental stage [12]. COX-2 is overexpressed in human GC cells and associated with poor overall survival [13,14]. Selective cyclooxygenase-2 (COX-2) inhibitors suppress the proliferation and induce the apoptosis of GC cells [15]. In the present study, two human GC cell lines BGC-823 and SGC-7901 were applied to assess the anti-tumor effects of myrrh on human gastric cancer. In addition, the expressions of COX-2, proliferating cell nuclear antigen (PCNA), and apoptosis-related proteins were detected to further elucidate the hidden mechanism.

## Materials and methods

### Water decocting extract of myrrh

Myrrh (#71202500) was purchased from Jiangsu Province Hospital of Traditional Chinese Medicine (Nanjing, China). The extract of myrrh was prepared as previously described [16,17]. Powdered myrrh resin (1.0 kg) was extracted with enough solvent (2 × 10 L) lasting for 1 h. The extraction process was repeated twice. Then, the extracted solution was boiled in a reflux condensation device for 2 h, naturally cooled to room temperature, and centrifuged at 3500 rpm for 20 min to remove the residues. Then, the supernatant was evaporated in a rotary evaporator for 12 h to obtain the myrrh powder. To prepare the decoction of myrrh extracts, 100 mg of myrrh powder was dissolved in 5 ml PBS (for the *in vivo* experiment) or cell culture medium (for the *in vitro* experiment) in a water bath at 90°C–100°C for 12 h. The mixture was centrifuged at 10,000 rpm for 20 min (twice) to obtain a supernatant, passed through a 0.22 μm filter, and stored at 4°C.

### Cell lines and cell culture

Poorly differentiated BGC-823 and moderately differentiated SGC-7901 human cell lines were obtained from Shanghai Institute of Cell Biology (Shanghai, China). All cells were routinely cultured in RPMI 1640 medium (BioInd, Israel) supplemented with 10% fetal bovine serum (FBS) and 1% penicillin/streptomycin (Gibco, Carlsbad, CA, U.S.A.). All the cells were kept at 37°C in a humidified atmosphere of 5% CO_2_ incubator.

### MTT assay

The effect of myrrh on GC cells viability was determined using 3–(4,5–dimethylthiazol–2–yl)-2,5–diphenyltetrazolium bromide (MTT, Sigma) assay. BGC-823 and SGC-7901 cells were seeded into 96-well microplate (4 × 10^3^ cells per well) and incubated overnight in 10% FBS medium. After 24 h, the cells were incubated with different concentrations of myrrh (0, 0.5, 1, 1.5, 2, 2.5, and 3 mg/ml) for 12, 24, or 48 h at 37°C. Cell-free medium was used as blank control. Subsequently, 200 µl of MTT solution (0.5 mg/ml) was added to each well and incubated for 4 h at 37°C. Afterward, 200 µl of dimethyl sulfoxide (DMSO, Sigma) was added to each well. The proliferation-inhibitory effects of each combination were assessed using a microplate reader (MJ Research Inc.) at 570 nm [18].

### Flow cytometry analysis

The GC cells apoptosis was measured with flow cytometry using Annexin V, FITC Apoptosis Detection Kit (Dojindo, Japan). For each treatment, 2 × 10^5^ cells were harvested (0, 1, 1.5, and 2 mg/ml of myrrh for 24 h) and washed twice using a cold phosphate-buffered saline (PBS). Then, the cells were re-suspended in 0.6 ml of binding buffer and allowed to react with 10 µl of FITC-labeled Annexin V and 10 µl propidium iodide (PI) for 15 min at room temperature in the dark. Afterward, the cells were analyzed on a flow cytometer (Becton Dickinson, CA, U.S.A.). Apoptosis was assessed by Annexin V-FITC and propidium iodide staining.

### Hoechst 33342 staining

Morphological changes were demonstrated by fluorescent microscopy using Hoechst staining. BGC823 and SGC7901 cells were treated with different concentrations of myrrh (0, 1, 1.5, and 2 mg/ ml for 24 h), washed twice with PBS and fixed. After washing twice with PBS for 3 min, the cells were stained with 10 µg/ml Hoechst 33342 (Beyotime, China) for 5 min at room temperature and examined by fluorescence microscopy (Eclipse E-800; Nikon, Tokyo, Japan). The apoptotic cells were identified by nuclear fragmentation and chromatin condensation.

### *In vitro* wound healing assay

BGC823 and SGC7901 cells were seeded in six-well plates and cultured in an incubator until confluent monolayers formed. The cells were serum-starved for 12 h. Scratch wounds were created by scraping the cell layer across each culture plate using the tip of a 10 µl pipette. The debris was removed by washing the cells with PBS. At this starting time point (*t* = 0 h), wound margins were observed using phase contrast microscopy and photographed. Then, serum-free medium containing 0 or 1 mg/ml myrrh was added to the plates, and the cells were incubated for up to 48 h at 37°C. The same fields of the wound margin were photographed at 24 and 48 h.

### Xenograft assay

Athymic nude mice (4–6 weeks old and weighing 18–22 g) were obtained from and raised in specific pathogen-free (SPF) conditions at the Department of Laboratory Animal Center of Nanjing Medical University (Nanjing, China). All animal experiments were in compliance with the protocols set by Nanjing Medical University (NJMU) Institutional Animal Care and Use Committee, and performed at the Animal Center of Nanjing Medical University. Then, 0.1 ml of cell suspension containing 5–6 × 10^6^ BGC823 or SGC7901 cells was subcutaneously injected into the axillary space of the mice. At 7–8 days after implantation of the cells (when the tumor size was approximately 0.1–0.2 cm^3^), the mice were randomly divided into two groups (*n* = 5). Myrrh was administered daily at 80 mg/kg by oral gavage for 2 weeks. Control mice were treated with an equal volume of normal saline. The size of the tumor was measured every 2 days with a caliper, and the tumor volume was calculated using the following formula: (the shortest diameter)^2^ × (the longest diameter) × 0.5. At the end of treatment, nude mice were intraperitoneally injected with 3% pentobarbital sodium and were killed by excessive anesthesia with a dose of 90 ml/kg. The tumor tissue was weighed and excised for Western blot analysis. Results from each mouse were plotted as average tumor volume versus time.

### Western blot analysis

Western blot analysis was conducted as describing in our previous reports [19]. The cultured cells and tumor tissues were homogenized in RIPA lysis buffer (Biyuntian, China) with a protease inhibitor cocktail. Then, tissue or cell extracts were centrifuged at 12,000 r/min at 4°C, and supernatant fractions were collected. The total protein was determined with a BCA protein assay kit (Thermo Scientific, Rockford, IL, U.S.A.). Proteins samples were resolved on 10% SDS-PAGE gel and then transferred to polypropylene fluoride (Millipore, U.S.A.). The membranes were blocked in 5% non-fat dried milk containing 0.1% Tween-20 in TBST for 2 h at room temperature. Then, the membranes were incubated at 4°C overnight with the following primary antibodies. Antibodies: PCNA (1:4000, Abcam), Bcl-2 (1:1000, Abcam), Bax (1:2000, Abcam), COX-2 (1:2000, Abcam) and GAPDH (1:10,000, Sigma–Aldrich). After washing, the samples were incubated with HRP-conjugated goat anti-rabbit or anti-mouse IgG at room temperature for 1 h. Signals were detected using an enhanced chemiluminescence reagent (WBKLS0500, Millipore, U.S.A.). All the protein bands were quantified using densitometry image analysis software (Quantity One, Bio-Rad, Hercules, CA, U.S.A.). The relative expressions of PCNA, Bcl-2, Bax, and COX-2 were normalized to that of GAPDH.

### Statistical analysis

All data from at least three independent experiments were represented as mean ± standard deviation (SD) and statistically analyzed by two-tailed Student’s *t*-test or one-way analysis of variance (ANOVA) with Dunnett’s multiple comparison tests. Statistical analyses were performed using the GraphPad software package (GraphPad Software, Inc., U.S.A.). *P* <0.05 was considered statistically significant.

## Results

### Myrrh inhibited the proliferation of BGC-823 and SGC-7901 cells

**Figure 1:**
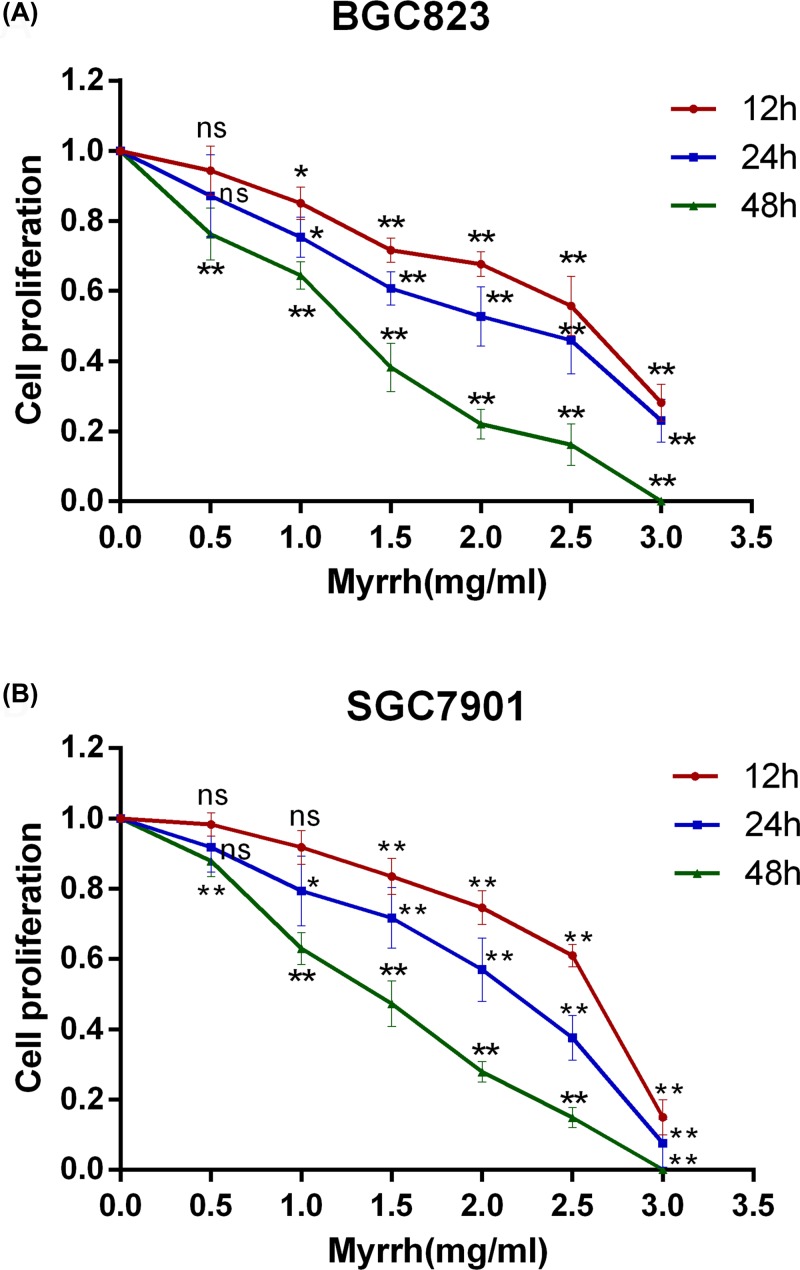
Myrrh inhibits GC cell proliferation (**A**) Dose- and time-dependent effects of myrrh on BGC-823 cell proliferation. (**B**) Dose- and time-dependent effects of myrrh on SGC-7901 cell proliferation. Each data point represents the mean ± SD from three independent experiments; ns (no significant), *P<0.05, **P<0.01 versus control (0 mg/ml).

MTT assay showed that myrrh significantly inhibited the proliferation of BGC-823 and SGC-7901 cells in a dose- and time-dependent manner (Figure 1). After 48 h of treatment with myrrh to 3 mg/ml, the proliferation of BGC823 and SGC7901 cells was almost completely blocked. The IC_50_ values of myrrh at different time points are shown in Table 1.

**Table 1:** IC_50_ values of myrrh at 12, 24, and 48 h on BGC-823 and SGC-7901 cells

IC50			
	Mean (SD)		
	12 h	24 h	48 h
BGC823	2.5017 (0.20)	2.0711 (0.26)	1.3578 (0.09)
SGC7901	2.4530 (0.05)	2.2118 (0.34)	1.4624 (0.03)

Data are represented as mean ± SD from three independent experiments

### Myrrh induced the apoptosis of BGC823 and SGC7901 cells

**Figure 2:**
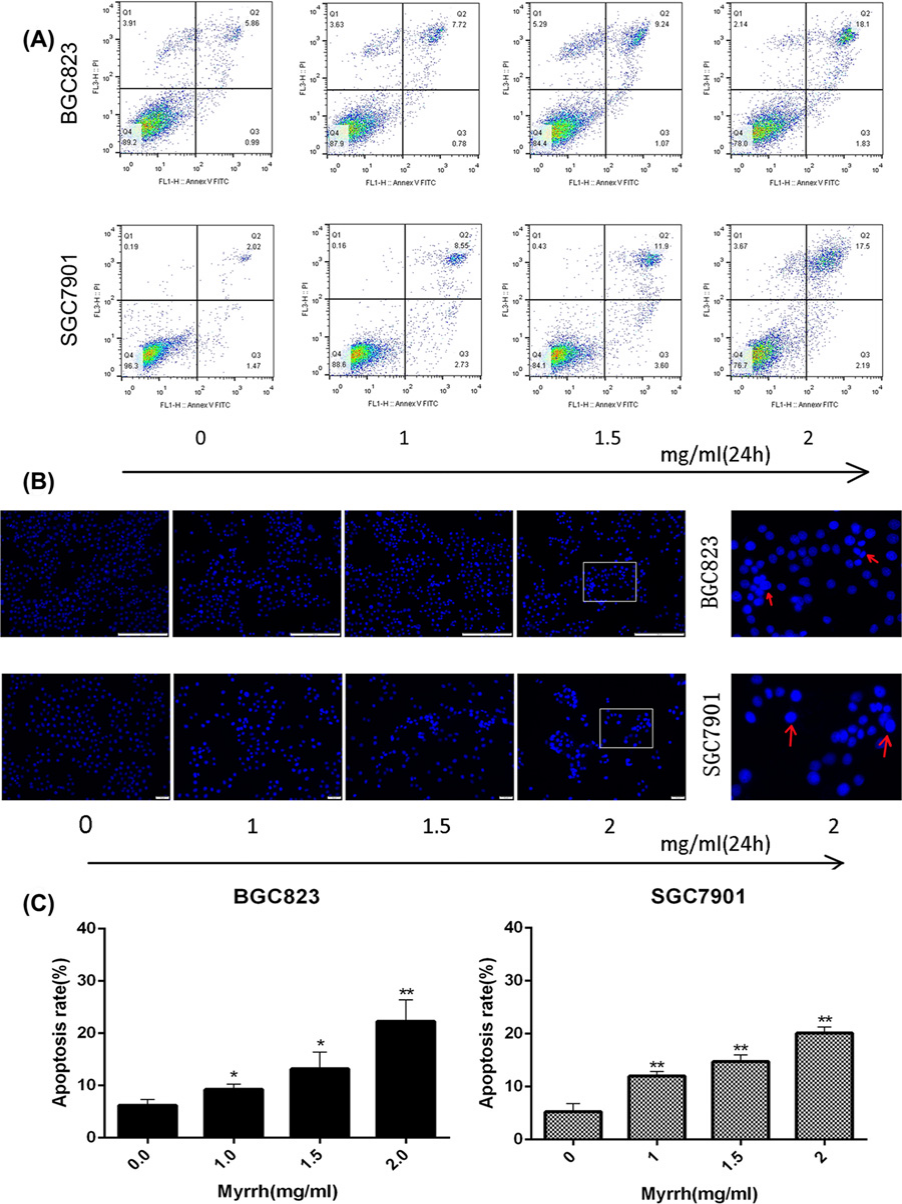
Myrrh induces apoptosis in GC cells (**A**) Flow cytometry-based annexin V-FITC/PI labeling of apoptotic cells. (**B**) Morphological changes in apoptotic cells were examined by fluorescence microscopy after Hoechst 33342 staining. Morphological changes were seen only in myrrh-treated gastric cancer cells. Myrrrh-treated BGC-823 and SGC-7901 cells clearly showed condensed and fragmented nucleus and emitted bright fluorescence. (**C**) The histogram represents apoptosis rates. Each data point represents the mean ± SD from three independent experiments; *P<0.05, **P<0.01 versus control (0 mg/ml)

AnnexinV-FITC/PI assay based on flow cytometry was carried out to investigate myrrh-induced apoptosis (Figure 2A). In the dual-parameter fluorescent dot plots, the cells in early apoptosis (Annexin V^+^/PI^−^, Q3) and late apoptosis (Annexin V^+^/PI^+^, Q2) were counted. Myrrh induced the apoptosis of both BGC-823 and SGC-7901 cells in a dose-dependent manner (Figure 2C). To confirm this effect, these myrrh-treated cells were identified by Hoechst 33342 nuclear staining. As shown in Figure 2B, the nucleus from the myrrh treated GC cells was condensed and fragmented, emitted bright fluorescence, which were early phenomena of apoptosis.

### Myrrh inhibited the migration of BGC-823 and SGC-7901 cells

**Figure 3:**
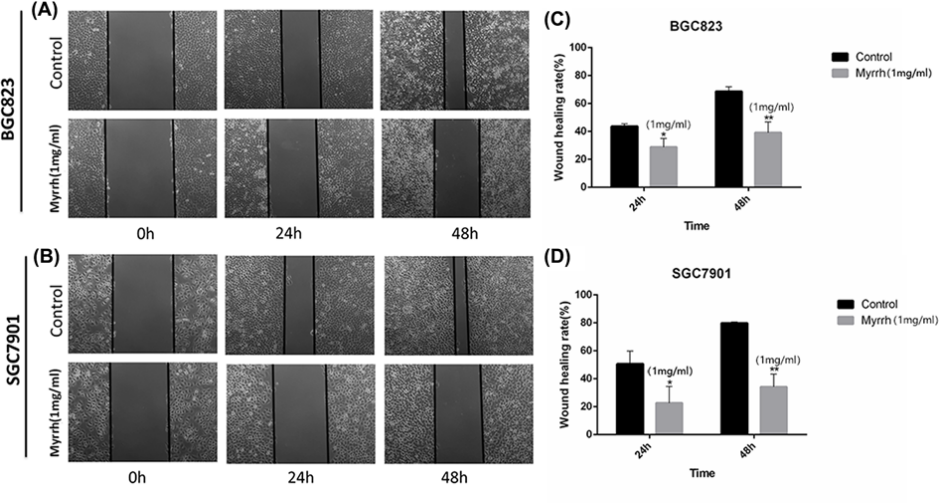
Myrrh inhibits monolayer wound healing of GC cells (**A** and **B**) Phase micrographs of BGC-823 and SGC-7901 cells at various times after monolayer wounding. (**C** and** D**) Quantification of cell migration using the monolayer wound healing assay. BGC-823 and SGC-7901 cells were treated without (control) or with 1 mg/ml Myrrh. Each data point represents the mean ± SD from three independent experiments; *P<0.05, **P<0.01 versus control (0 mg/ml).

The effect of 1 mg/ml myrrh on the migration of GC cells was investigated. After 24 and 48 h of myrrh treatment, the space between wounds widened (Figure 3), thereby indicating that healing was inhibited. The GC cells’ migratory capacity was weakened in myrrh-treated BGC-823 and SGC-7901 cells.

### Myrrh changed the expressions of PCNA, Bcl-2, Bax, and COX-2 in gastric cancer cells

**Figure 4:**
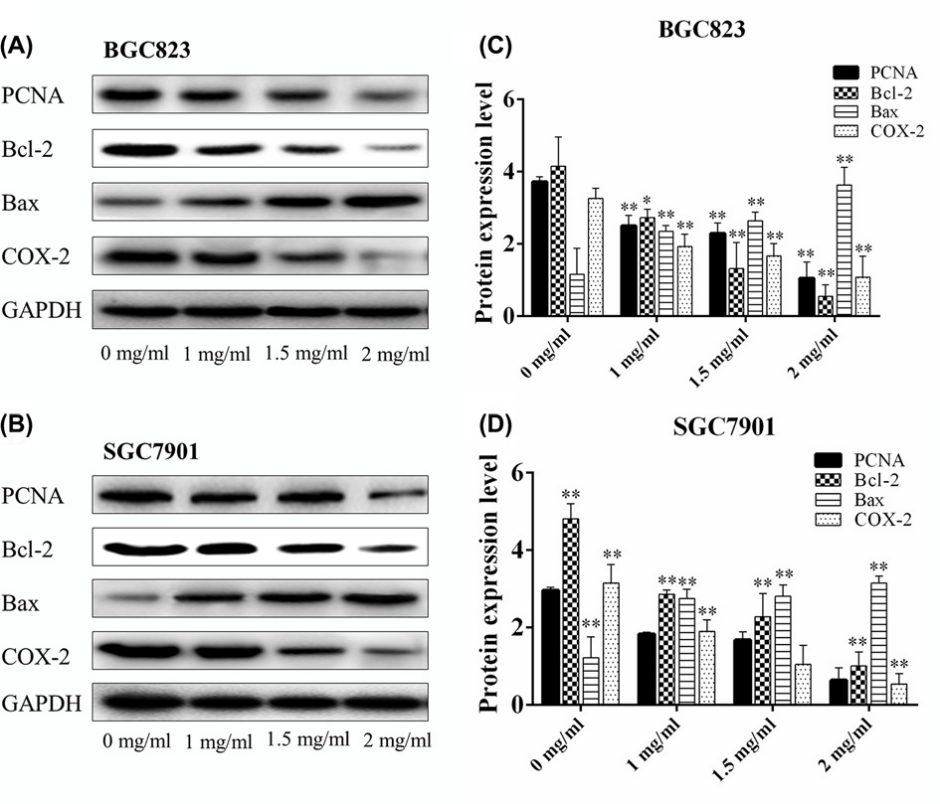
The effects of myrrh on expression PCNA, Bcl-2, Bax, and COX-2 levels (**A**) The expressions of PCNA, Bcl-2, Bax, and COX-2 in BGC-823 cells were measured by Western blot analysis after Myrrh treatment. (**C**) The histogram represents the relative expressions of PCNA, Bcl-2, Bax, and COX-2 compared with GAPDH in BGC-823 cells. (**B**) The expressions of PCNA, Bcl-2, Bax, and COX-2 in SGC7901 cells were measured by Western blot analysis after Myrrh treatment. (**D**) The histogram represents the relative expressions of PCNA, Bcl-2, Bax, and COX-2 compared with GAPDH in SGC7901 cells. Each data point represents the mean ± SD from three independent experiments; *P<0.05, **P<0.01 versus control (0 mg/ml).

The effects of myrrh on apoptosis-related proteins were evaluated by Western blot analysis. After 24 h of myrrh treatment, the expression of pro-apoptotic protein Bax was up-regulated and that of anti-apoptotic protein Bcl-2 was down-regulated in a dose-dependent manner in BGC-823 and SGC-7901 cells. In addition, myrrh significantly decreased PCNA and COX-2 expressions in a dose-dependent manner in BGC-823 and SGC-7901 cells (Figure 4).

### Myrrh inhibited gastric xenograft tumor growth *in vivo*

**Figure 5:**
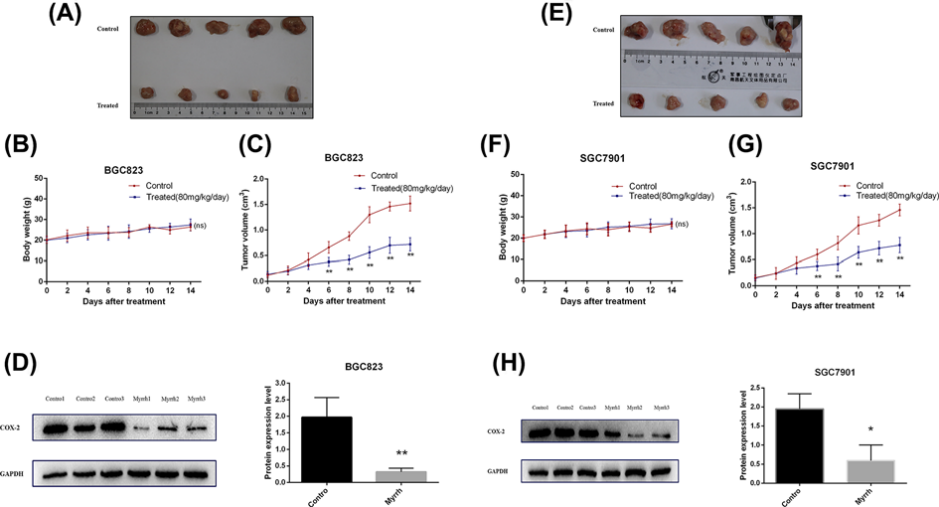
Myrrh inhibits gastric tumor growth *in vivo* The nude mice were subcutaneously injected with BGC-823 and SGC7901 cells. When the tumor size was approximately 0.1–0.2 cm^3^ in size, the mice were randomly divided into four groups (*n*=5) to receive different treatments. (**A** and** E**) Myrrh inhibits gastric xenograft tumor growth *in vivo*. (**B** and** F**) Body weight change of mice treated with saline or myrrh. (**C** and **G**) Tumor volume was measured, and the tumor growth curves were drafted. (**D** and** H**) COX-2 protein expression in tumor tissues from myrrh-treated and control mice. Data were shown as mean ± SD from five mice; *P<0.05, **P<0.01 versus control (saline)

The effect of myrrh on BGC823 and SGC7901 cell xenografts was examined (Figure 5). The mice implanted with GC cells were treated daily with myrrh for 2 weeks. No death occurred within 2 weeks. There was no significant difference in body weight between the control and myrrh-treated nude mice groups (Figure 5B,F). The average tumor volume of nude mice treated with myrrh was markedly lower than that in the control nude mice (Figure 5C,G).

### Myrrh down-regulated the expression of COX-2 in the GC xenograft model

As shown in Figure 5D,H, COX-2 protein was overexpressed in control nude mice’s GC tissues. Intragastric administration of myrrh reduced the overexpression of COX-2 in the tumor tissues of nude mice, thereby suggesting that myrrh may inhibit the growth of tumors in nude mice by down-regulating the expression of COX-2 in GC tissues.

## Discussion

GC is a digestive malignancy with a high incidence and mortality globally [20]. Despite therapeutic improvement, it remains a leading cause of cancer-associated death [21]. GC can be diagnosed early by endoscopy and treated by timely surgical excision, but the patient’s long-term survival is still threatened by high metastasis, recurrence, and drug resistance [22–24]. Active medicinal compounds from natural sources have provided alternative treatment options. Myrrh, resinous exudate of *Commiphora Myrrha* (Nees) *Engl*, shows anti-tumor properties *in vivo* and *in vitro* [6,9,25]. In the present study, we explored for the first time the effects of myrrh on the proliferation, apoptosis, and migration of GC cells.

COX-2, an enzyme undetectable in most normal tissues, is induced by various cytokines, hormones, and growth factors, and is highly expressed in inflammation and tumor tissues [26–28]. COX-2 causes malignancy by up-regulating the production of prostaglandins, primarily prostaglandin E2 (PGE2). Over-expressed COX-2 participates in invasion and metastasis and aggravates the prognosis of GC through multiple pathways [29,30]. COX-2 is a potential anti-cancer target molecule for the treatment and prevention of malignant tumors [31]. Myrrh can inhibit the proliferation of head and neck cancer cells by reducing the expression of COX-2 [32]. The present study is the first to investigate the effect of myrrh on the proliferation and apoptosis of GC cells. We showed that myrrh significantly lowered the expression of COX-2 in BGC-823 and SGC-7901 cells.

Tumorigenesis results from the imbalance between cell proliferation and apoptosis [33]. To clarify the molecular mechanisms of myrrh in gastric tumorigenesis, the expressions of proteins associated with cell proliferation and apoptosis were examined *in vitro*. A tumor mouse model was constructed to observe the anti-tumor effect of myrrh *in vivo*. PCNA, a protein essential for DNA replication and damage and repair, is an efficient marker to assess the growth fraction of a cell colony [34,35]. Our experiments demonstrated that myrrh dose-dependently reduced PCNA expression in BGC-823 and SGC-7901 cells. Bcl-2 family proteins are involved in the anti-apoptotic machinery and overexpressed in different malignancies [36]. Bcl-2 and related cytoplasmic proteins are key regulators in cell apoptosis. They are to inhibitory (Bcl-2, Bcl-XL, Bcl-w, A1, and Mcl-1) or acceleratory (Bax, Bim, Bak, and Bcl-XS) [37]. Down-regulation of COX-2 expression can induce cancer cell apoptosis by down-regulating Bcl-2 expression and up-regulating Bax expression [38,39].

**Figure 6:**
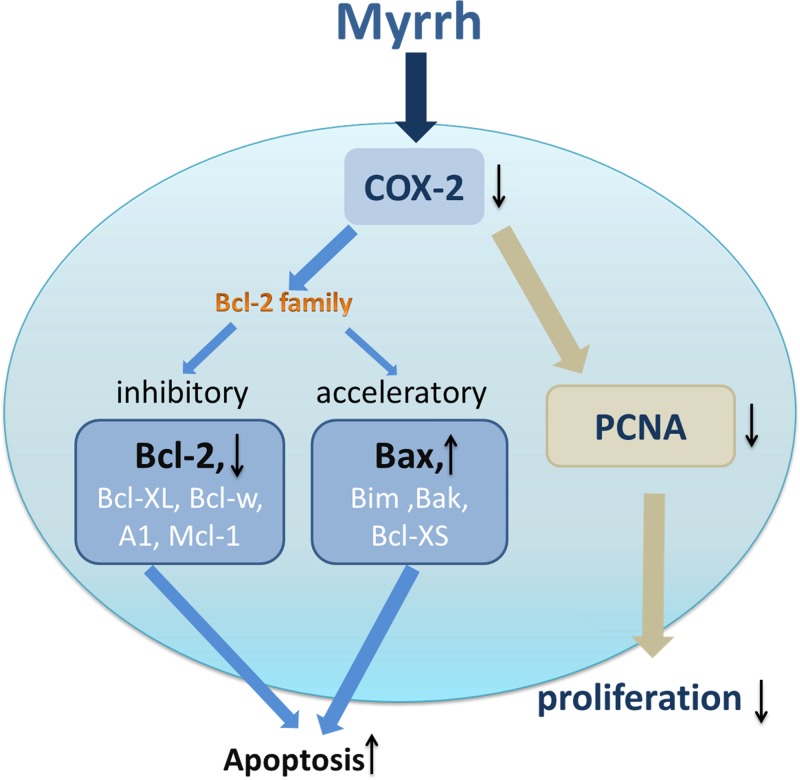
Hypothetical model of Myrrh’s inhibition of proliferation of gastric cancer cells and induction of apoptosis

Our previous studies have demonstrated that harmine can down-regulate PCNA and COX-2 expression in human gastric cancer cells. In addition, harmine significantly increases Bax protein level and decreases the Bcl-2 protein level in human GC cells [40]. Our results are consistent with previous research, and suggest that myrrh inhibits the proliferation and induces the apoptosis of GC cells probably by down-regulating COX-2 expression in GC cells. A schematic diagram of the relevant protein of interest (Figure 6) showed the simultaneous up-regulation of Bax expression and down-regulation of Bcl-2 expression and COX-2 in myrrh-treated GC cells. Results of the *in vitro* study provide evidence that myrrh induces the apoptosis of GC cells in a dose-dependent manner. Myrrh could reduce GC cell migration rate *in vitro*. Moreover, with increasing administrative time, myrrh’s inhibition of GC cell migration may increase.

*In vitro* data suggested that myrrh suppressed GC cells growth by inducing the apoptosis of GC cells. Our animal experiments verified the findings of *in vitro* experiments. Previous research confirmed that myrrh (or guggulsterone) has an anti-tumor effect on mice [17,41,42]. The tumor size was significantly smaller in myrrh-treated animals compared with that in the control group. Myrrh significantly inhibited tumor growth in xenograft nude mice without causing significant weight loss, mortality, or other side effects.

In conclusion, our experiments demonstrated for the first time that myrrh can inhibit the growth of human GC cells by down-regulating COX-2 expression *in vitro* and *in vivo*. Myrrh is a potential therapeutic agent for GC.

## Abbreviations

COX-2: cyclooxygenase-2
GC: gastric cancer
PCNA: proliferating cell nuclear antigen
PGE2: prostaglandin E2
